# Drought mediated physiological and molecular changes in muskmelon (*Cucumis melo* L.)

**DOI:** 10.1371/journal.pone.0222647

**Published:** 2019-09-24

**Authors:** Waquar Akhter Ansari, Neelam Atri, Javed Ahmad, Mohammad Irfan Qureshi, Bijendra Singh, Ram Kumar, Vandna Rai, Sudhakar Pandey

**Affiliations:** 1 ICAR–Indian Institute of Vegetable Research, Varanasi, Uttar Pradesh, India; 2 Department of Botany, M.M.V., Banaras Hindu University, Varanasi, Uttar Pradesh, India; 3 Proteomics & Bioinformatics Lab, Department of Biotechnology, Jamia Millia Islamia, New Delhi, India; 4 ICAR–National Research Centre on Plant Biotechnology, LBS Centre, Pusa Campus, New Delhi, India; Huazhong Agriculture University, CHINA

## Abstract

Water deficiency up to a certain level and duration leads to a stress condition called drought. It is a multi-dimensional stress causing alteration in the physiological, morphological, biochemical, and molecular traits in plants resulting in improper plant growth and development. Drought is one of the major abiotic stresses responsible for loss of crops including muskmelon (*Cucumis melo*. L). Muskmelon genotype SC-15, which exhibits high drought resistance as reported in our earlier reports, was exposed to deficient water condition and studied for alteration in physiological, molecular and proteomic profile changes in the leaves. Drought stress results in reduced net photosynthetic rate (Pn), stomatal conductance (Gs) and transpiration (E) rate. With expanded severity of drought, declination recorded in content of total chlorophyll and carotenoid while enhancement observed in phenol content indicating generation of oxidative stress. In contrary, activities of catalase (CAT), superoxide dismutase (SOD), ascorbate peroxidase (APX), and guaiacol (POD) were increased under drought stress. Peptide mass fingerprinting (PMF) showed that drought increased the relative abundance of 38 spots while decreases10 spots of protein. The identified proteins belong to protein synthesis, photosynthesis, nucleotide biosynthesis, stress response, transcription regulation, metabolism, energy and DNA binding. A drought-induced MADS-box transcription factor was identified. The present findings indicate that under drought muskmelon elevates the abundance of defense proteins and suppresses catabolic proteins. The data obtained exhibits possible mechanisms adopted by muskmelon to counter the impacts of drought induced stress.

## Introduction

Muskmelon (*Cucumis melo* L.) is a tropical old-world cucurbit species. It belongs to Cucurbitaceae, mostly commercially cultivated in arid and semi-arid regions, areas often suffering rainfall limitation [1, 2] According to FAOSTAT database in the year 2018 worldwide 29.39 million tons melons were produced, India ranks fifth with 1.03 million tons production [3]. Under drought stress, loss in muskmelon productivity recorded from 25–30 t/ha to 12–15 t/ha in India, however, it was 50% and 23% in China and Egypt, respectively [4, 5].Potential novel alleles that muskmelon possess can be exploited for biotic and abiotic stresses tolerance, also yield and other related traits. Indian melon landraces distributed across numerous ecological niches, which includes arid to humid tropical environments. Resistant against various abiotic stresses in Indian melon landraces were reported [1].

During their growth period, plants might face many harsh environments, and respond through various biochemical, physiological, and molecular mechanism, which is tackled by acquiring tolerance through a wide range of traits [6]. Overall, acclimatization to drought stress is mediated via subtle alteration in gene expression which leads to changes in composition of plant transcriptome, proteome and metablome; ultimately, phenotype [7, 8, 9, 10, 11, 12, 13]. Since, plants react to drought through different strategy; as a result, single mechanism is not sufficient to describe or advance tolerance against drought in plants [14]. Therefore, study on the proteome changes due to water-deficit will be a potent tool for understanding mechanisms of drought tolerance in plants. Recently many reports on changes in plant proteome under abiotic stress in large number of plant species have been published. Successful proteomics study for drought stress were reported in wheat [15]; *Brassica napus* [16]; maize [17]; barley [18] and wild watermelon [19]. Under drought stress plants expresses different proteins, not specifically related to drought, other than induced due to the damages of cell. Which comprise diverse group of heat shock protein (HSPs) related genes, proteinase inhibitors, thiol proteases, and osmotin in plants. Changes in protein accumulation under stress are strongly interrelated to response of plant through phenotypic changes which influence plant tolerance against stress.

Many proteomics study report on melon are available for different traits [20, 21], but report on proteomics study for drought stress is still lacking for muskmelon. Therefore, present investigation was undertaken to study the changes in muskmelon proteome under drought stress. The study has high importance, since proteins, unlike transcripts, are ultimate players of plant stress response. The objectives included investigation of physiological, biochemical, antioxidant activities and proteomic response of muskmelon (genotype SC-15) to drought stress. The muskmelon genotype SC-15 was identified as drought tolerant genotype [10, 12]. Muskmelon (SC-15) was exposed to drought stress and changes in protein profile were explored through two-dimensional gel electrophoresis (2-DE) followed by MALDI-TOF mass spectrometry.

## Materials and methods

### Plant materials and stress conditions

The muskmelon genotype SC-15 seeds were obtained from ICAR-Indian Institute of Vegetable Research, Varanasi, Uttar Pradesh, India, and used in present study. Plants were raised in a greenhouse between the periods of March to the mid of the May 2014–2016 with controlled environment: 42/22 °C (day/night), relative humidity (60–70%), photoperiod (14 h), with photosynthetically active radiation of 1000 μmol m^-2^ s^-1^. Muskmelon seeds were germinated in trays containing coco peat and vermiculite mixture. Plants were grown till attaining true leaf stage, transplanted one each in 40 pots, the diameter and height of the pots was 22 cm and 23.8 cm respectively. Each pot contained sand, loamy clay and farmyard manure in a mixture of 1:2:1 ratio, pH of the pot soil was 6.6 while its bulk density was of 1.34 g/cm^3^. On every third day, each pot was supplied with 1.5 L of autoclaved tap water till 25 days after day of germination (DAG). Then, pots were equally distributed in 4 sets: Set 1: exposure of 21 days drought, Set 2: 14 days of drought, Set 3: 7 days of drought, and Set 4: 0 days of drought. The average soil water content (SWC) in pots were approximately 8%, 14%, 27% and 46% respectively for the set 1, set 2, set 3 and set 4, respectively as estimated by method of Coombs *et al*. [22]. Leaf samples were harvested from each treatment and instantly kept in liquid nitrogen and preserve at -80 °C in anticipation of their analysis further. The experiments were carried out in three different biological replicates.

### Dry and fresh weight ratio

The plants part was separated into root, shoot and leaf and their fresh weights were measured immediately. Later, samples dried at 65 °C for 72 h in a hot-air oven and measurements of their dry weight done. The ratios of dry and fresh weights were calculated as per the [23] formula with slight modification:

RDW/RFW%=(RDW/RFW)×100

SDW/SFW%=(SDW/SFW)×100

LDW/LFW%=(LDW/LFW)×100

Where, RDW, RFW, SDW, SFW, LDW and LFW, represents, root dry weight, root fresh weight, shoot dry weight, shoot fresh weight, leaf dry weight and leaf fresh weight, respectively.

### Gas exchange and chlorophyll fluorescence

Portable photosynthetic system (Model LI 6400, LICOR, Lincoln, Nebraska, USA) were used for the measurement of Photosynthetic rate (Pn), stomatal conductance (Gs) and transpiration (E), timing of data recording was between 11:00 AM to 1:00 PM using 250 cm^3^ closed circuit cuvette [24]. Respective specifications for data recording was as follows: 350 mmol mol^-1^ ambient CO_2_ concentration, cuvette air temperature was set at 32 °C before and after the relative treatments, and at 28 °C during the relative treatment, and relative humidity 65–70%. A portable Handy Plant Efficiency Analyzer (Hansatech Instruments, King’s Lynn, Norfolk, United Kingdom) were utilized for the measurement of photosynthetic efficiency. First, plant leaves adapted for 30 min. using leaf clips for dark condition towards adaxial side. Surface of the leaves were irradiated exposing red light, and the generated signal of fluorescence at excitation irradiance was collected, which was positioned at 3000 μmol m^−2^ s^−1^commencing from the consistent leaf surface. Minimum (*F*_*0*_) and maximum (*F*_*m*_) chlorophyll fluorescence recorded and maximum quantum efficiency of photo system II calculated according to the formula *F*_*v*_/*F*_*m*_ = (*F*_*m*_-*F*_*0*_)/*F*_*m*_ [25].

### Photosynthetic pigment and total phenols

For chlorophyll and carotenoid estimation, 300 mg leaf tissues were taken for crushing in chilled acetone (80%) using a mortar and pestle. At 663, 645 and 470 nm supernatant absorbance was recorded and as per [26] concentration calculated. For different chlorophyll pigments and carotenoid contents (mg g^-1^ fresh weight) measured as per following formula:

chlorophyll a (mg g^-1^) = [(12.7 × A_663_−2.69 × A_645_)]

chlorophyll b (mg g^-1^) = [(22.9 × A645−4.68 × A_663_)]

carotenoid (mg g^-1^) = [{(1000 × A_470_)—(3.27 × chlorophyll a + chlorophyll b)}/227].

Earlier described method of [27] was employed to measure the phenol content in leaves. In 5 mL of 80% methanol leaves tissue (500 mg) were homogenized and left in a water bath for 15 min at 70 °C. The absorbance recorded at 725 nm.

### Gene expression analysis

For RT-qPCR, eight primers designed using the gene sequence responsible to encode the proteins identified through MALDI-TOF, the sequences of designed primers are presented in Table 1. In addition to these primers, antioxidative enzymes, superoxide dismutase (*Cyt-SOD*), catalase (*CAT*), ascorbate peroxidase, (*APX*) and glutathione reductase (*GR*), gene specific primers reported previously were used to study the respective gene expression changes under progressive water-deficit condition [28], sequence information is given in S1 Table. TRI reagent (Ambion) in combination with RNAase-free DNAase treatment (Qiagen, USA) were used for total RNA extraction. According to the manufacturer instructions cDNA synthesis kit (Bio-Rad, USA), were used for first-strand cDNA synthesis, for this in 20.0 μL reaction volume, 1 μg of total RNA was used. As per manufacturer’s instructions IQ SYBR Green Supermix (Bio-Rad, USA) were used for expression analysis, using iQ5 thermo cycler (Bio-Rad, Hercules, CA, USA) with iQ5 Optical System Software version 2.0 (Bio-Rad, Hercules, CA, USA).

**Table 1:** The homologue proteins, spot number, primer sequence, melting temperature, product size and fold change in gene expression, are given for muskmelon plants grown under 0 (well-watered) conditions over plants grown under 7 and 14 days of water-deficit.

S.No	Homologue Proteins	*Spot No.	Primer Sequence	Tm	Product size	Relative fold change in gene expression	
						7 days	14 days
1	RNA pseudouridine synthase 7	4	F-GCCTTGTCTCAGGACTTCTTATC R-ACCCACTACCCTTGCAATATAC	62	113	2.03	13.5
2	Ras-related protein RABD1	10	F-CTGTGGAACTGGATGGAAAGA R-TCCATGTGCACCTCTGTAATAG	62	104	8.75	7.66
3	MADS-box transcription factor	25	F-GAACCAAGATAGCGGAAGTAGAG R-GTCCAGCAGGTTCCATGATATT	62	132	1.96	0.31
4	Chromoplast-specific carotenoid-associated protein	39	F-CTCTAGCCACCACTTCCATTAC R-GGTGTTCCAATGACACCTTCT	62	97	2.01	9.65
5	Dihydroflavonol-4-reductase	41	F-CGTCAAGATGACAGGATGGATG R- GGATGATGCTGATGAAGTCCAG	62	104	6.32	11.75
6	28 kDa ribonucleoprotein	42	F-GAGCCAAACGAAGATGCTAAAC R-CACAGTTCCAGCCTTCTCAA	62	96	7.39	13.78
7	Cytochrome P450 CYP93B25	46	F-TCATTGGCCATCTCCATCTC R-CGCAAGGGACTGATCCTAAA	62	108	2.27	1.3
8	NADPH adrenodoxin oxidoreductase	48	F-AGGAATCGTACCCAACATTAGAG R-CTCTTCAACCACCCACATACA	62	99	0.73	1.21

*Gene ID for respective spot number are in Table 3

### Protein extraction

Trichloroacetic acid (TCA)/acetone method was used for total soluble protein extraction [29]. Frozen leaf material was crushed using liquid nitrogen, powder obtained were collected in 40 mM Tris-Base (pH 7.5), EDTA (2 mM), 2% (w/v) PVP, β-mercaptoethanol 0.07% (v/v), Triton X100 1% (v/v), PMSF (1 mM) and glycerol 10% (v/v), vortexed and left to room temperature for 2 h, then at 4 °C for 15 min centrifuged at 15,000 rpm. In 10% cold TCA the extract was mixed, additionally β-mercaptoethanol 0.07% (v/v) and EDTA (2 mM) was added in the supernatant, finally kept for overnight at -20 °C. The mixtures were centrifuged at 5000 rpm for 5 min, continued by chilled acetone thrice gentle washings. Obtained pellet was vacuum dried and resuspended in solubilization buffer consisting of urea (7 M), thiourea (2 M), CHAPS (4%), DTT (40 mM) and ampholytes (0.2%) (Ampholine, Bio-Rad, USA) at pH 3–10, followed by centrifuge at 6000 rpm for 5 min at room temperature. Total protein content was quantified by standard method using [30]. Bovine serum albumin (BSA) served as the protein standard.

### Two-dimensional gel electrophoresis and image analysis

Performed two-dimensional gel electrophoresis applying standard procedure. With 150 mL of rehydration buffer immobilized pH gradients (IPG) strips (11 cm, pH 4–7, linear, Bio-Rad, USA) rehydrated overnight at room temperature in a re-swelling tray (Bio-Rad, USA). An amount of 250 μg protein was focused (1^st^D) on immobilized pH gradient (IPG) strips. The operating condition for isoelectric focusing (IEF) was as follows: 250 V for 1 h, 500 V for 1 h, 1000 V for 2 h, and 2000 V for 2 h and 3641 V for 2 h for a total of 65000 V, in an equilibration buffer A [Tris-HCl (50 mM), pH 8.8, urea (6 M), glycerol 30% (v/v), SDS 2% (w/v), with small amount of bromophenol blue] including DTT (10 mg/mL). IPG strips were equilibrated after IEF run for 15 min followed by equilibration buffer B [Tris-HCl (50 mM), pH 8.8, urea (6 M), glycerol 30% (v/v), SDS 2% (w/v), and a small amount of bromophenol blue] containing iodoacetamide (25 mg/mL), for protein reduction and alkylation, respectively. Further, each IPG strip with focused protein were kept on upper part of the SDS-PAGE gel and sealed with 0.8% agarose. Protein was resolved at 120 V at 15 °C using mixed protein ladder. Staining of gels were performed using Coomassie Brilliant Blue [31] stain and photographed using Gel-Doc (Bio-Rad, USA).

### In-gel digestion of proteins

As per protocol given by [32], selected proteins in-gel tryptic digestion performed. Selected protein spots were excised manually and transferred into 0.2 mL microcentrifuge tube. Using ultra-pure water (100 μL) gel slices were washed and centrifuged for 20 min at 1100 rpm, and de-stained with 50% (v/v) acetonitrile (ACN). Same process was repeated using 100 μL of ACN (100% v/v), 45 μL of ammonium bicarbonate (NH_4_HCO_3_) and 5 μL of DTT (1 M) were added to the gel slices and incubated for 45 min at 65 °C. 100μL of iodoacetamide (0.1 M) in NH_4_HCO_3_ (50 mM) were added and centrifuged for 30 min at 22 °C at 1100 rpm. The trypsin (Promega, USA) was dissolved in a concentration of 1 μg/μL in hydrochloric acid (1mM), further diluted to 20 μg/mL in NH_4_HCO_3_ (50 mM). The gel slices were incubated in a minimum volume of the trypsin solution (20 μL), and left in ice for 45 min. then 50 μL of NH_4_HCO_3_ (50 mM) was added and kept for overnight (12–16 h) at 37 °C. Five μL of 1% TFA was added and supernatant was transferred. In gel slices, 100 μL of ACN (50%) and 5% TFA (Trifluoroacetic acid) were added, and incubated at room temperature for 1 h. Pooled extracts were freeze dried using lyophilizer (Labconco, USA), and shifted in -80 °C for further mass spectrometry study and peptide mass fingerprinting (PMF).

### MALDI–TOF–MS

Resuspended an equal volume of digested proteins in acetonitrile (50%) and trifluoroacetic acid (0.1%). To prepare the sample, 0.5 μL of samples were mixed with 0.5 μL of matrix solution (5 mg/mL a-Cyano-4-hydroxycinnamic acid (CHCA) in ACN (50%) containing TFA (0.1%)). Finally the prepared samples were dropped on MPT 384 plate ground steel (Bruker Daltonics, Germany, part no. 209519), spotted samples were left for air-dry at room temperature. The PMF of differentially expressed proteins was recorded by employing Ultraflex MALDI-TOF/TOF MS analyzer (Bruker Daltonics, Billerica, MA, USA). On the basis of PMF, protein identification was performed through exploring non-redundant (NR) protein database of NCBI (http://www.ncbi.nlm.nih.gov) by means of monoisotopic peaks with the MASCOT (Matrix Science, UK), search engine (http://www.matrixscience.com) [33], with taxonomy of Viridiplantae (Green Plants). As per [34] suggested, the identified proteins with relatively significant score were taken for consideration and additionally proteins functional categorization was performed. To evaluate the protein identification, hits with greater than 15 MOWSE score was considered significant by searching NCBI, NR database of protein [35].

### Statistical analyses

The data were analyzed and presented as mean with standard error of at least three independent replications. For this a simple variance analysis (ANOVA) at (P≤ 0.05) using SPSS 16.0 (SPSS, Inc., Chicago, USA) was performed. When ANOVA were found significant the Duncan multiple range test of the data was done for the mean values comparison.

## Results

### Effect of water-deficit on growth of melon plants

**Fig 1:**
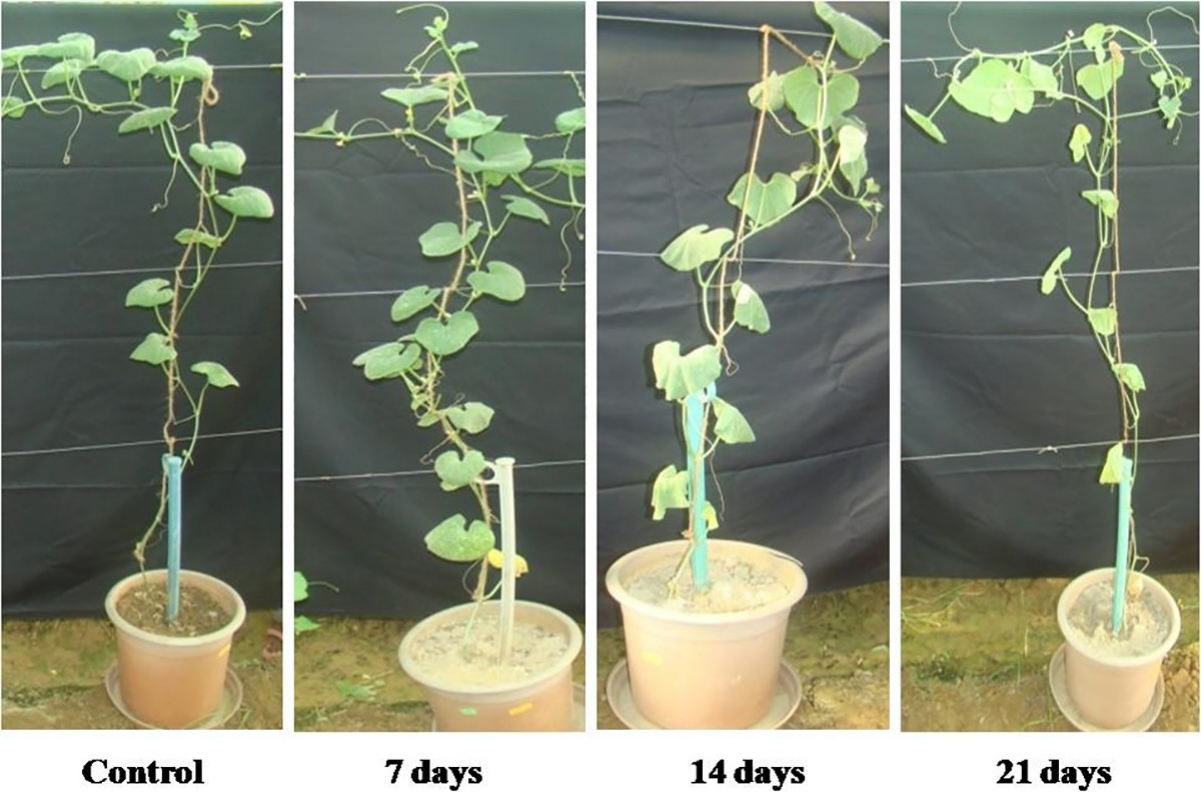
Effect of water-deficit treatments on drought tolerant muskmelon genotype SC-15 plants. Under 0 (well-watered), 7, 14 and 21 days of water-deficit.

The leaf area, root volume, branching of the roots along with shoots and roots length was assessed under different water-deficit treatments (Table 2). The growth of shoots was inhibited significantly with the increase in days of water-deficit (DWD) (Fig 1). As a result of which leaf area, root volume and root branching was significantly lower than values of well-watered i.e. controlled plants. However, a prominent increase in the root length after 7, 14 and 21 DWD was noted. The decrease in the leaf area was by 17%, 25.9% and 36%, after 7, 14 and 21 DWD, respectively, in comparison to control. There was a decrease in root branching by 46% after 21 DWD, while it was 33% and 14% after 14 and 7 DWD, respectively, (Table 2). Root volume and shoot length was reduced by 8%, 13%, 17% and 17%, 38% and 44%, respectively, after 7, 14 and 21 DWD, compared with control values. While, there was an increase in root length by 22%, 39% and 55%, respectively, under similar water-deficit condition. Dry biomass dry and fresh weight ratio was positively affected due to progressive water-deficiency, as significant increase in DRW/FRW, SDW/SFW and LDW/LFW was recorded after 7, 14 and 21 DWD. Maximum increase in DRW/FRW, SDW/SFW and LDW/LFW was observed after 21 DWD, with respective values of 38%, 51% and 40%, while it was minimum at 15%, 8% and 13% increase after 7 DWD. However, after 14 DWD, 28%, 27% and 27% increase was recorded, respectively, compared with control.

**Table 2:** Various growth related and physiological parameters of muskmelon plants observed under 0 (well-watered), 7, 14 and 21 days of water-deficit.

Parameters observed	Days of drought stress			
	*0 days	7 days	14 days	21 days
RDW/RFW (%)	13.6 ± 1.11^c^	15.9± 1.13^bc^	18.8 ± 1.5^ab^	22 ± 1.26^a^
SDW/SFW (%)	10.8 ± 0.3^c^	11.7 ± 0.72^bc^	14.8 ± 1.34^b^	22.1 ± 1.33^a^
LDW/LFW (%)	20.8 ± 1.76^b^	23.9 ± 1.91^b^	28.5 ± 3.04^ab^	34.4 ± 2.8^a^
Root branches (N)	14 ± 1.16^a^	12.1 ± 1.21^ab^	9.34 ± 0.22^bc^	7.6 ± 0.42^c^
Root girdth (cm)	3.27 ± 0.16^a^	3.02 ± 0.10^a^	2.83 ± 0.26^a^	2.72 ± 0.13^a^
Root length (cm)	24.5 ± 1.95^d^	31.5 ± 1.62^c^	40.7 ± 1.88^b^	54.6 ± 2.9^a^
Shoot length (cm)	107 ± 7.19^a^	88.5 ± 4.37^b^	65.52± 5.61^c^	59.9 ± 2.34^c^
Leaf Area (cm)	133 ± 6^a^	110 ± 8.74^ab^	98.5 ± 7.08^bc^	85.2 ± 6.37^c^
Photosynthetic rate (μmol m^-2^ s^-1^)	14.3 ± 1.2^a^	12.1 ± 1.4^ab^	11 ± 1.0^ab^	9.1 ± 1.27^b^
Stomatal conductance (mmol m^-2^ s^-1^)	1.15 ± 0.04^a^	1.04 ± 0.05^a^	0.86 ± 0.04^b^	0.81 ± 0.06^b^
Transpiration rate (mmol m^-2^s^-1^)	13.7 ± 1.02^a^	11.2 ± 1.15^ab^	8.76 ± 0.60^b^	5.38 ± 0.32^c^
*Fv*/*Fm*	0.75 ± 0.02^a^	0.73 ± 0.02^a^	0.64 ± 0.02^b^	0.57 ± 0.01^c^

RDW; Root Dry Weight, RFW; Root Dry Weight, SDW; Shoot Dry Weight, SFW; Shoot Fresh Weight, LDW; Leaf Dry Weight, LFW; Leaf Fresh Weight, N; Number, cm; centimeter. The results are mean ± SE of triplicate measurements. Means followed by the same letter along same row are not significantly different (P ≤ 0.05), according to Duncan’s multiple range test.

*0 days—control

### Gas exchange and chlorophyll fluorescence

The gas-exchange parameters of photosynthesis (Pn), stomatal conductance (Gs) and transpiration (E) rates decreased with increase in DWD (Table 2). The decrease in Pn was observed of 15%, 23% and 36% after 7, 14 and 21 DWD, respectively in comparison to the well-watered plants. Similar trend was also observed in stomatal conductance (Gs) and transpiration (E). Stomatal conductance (Gs) decreased by 9.6%, 25.2%, 29.6% while transpiration (E) decreased by 18%, 36%, and 61%, compared with control, after 7, 14 and 21 DWD, respectively. The decrease in E was drastic by more than 60% after 21 DWD (Table 2). *Fv*/*Fm* was measured in the leaves adapted to dark, showing a reduction of 3% after 7 DWD. However the decline in *Fv*/*Fm* was higher after 14 and 21 DWD, by 14% and 23% compared with control (Table 2).

### Phenol and pigment content

**Fig 2:**
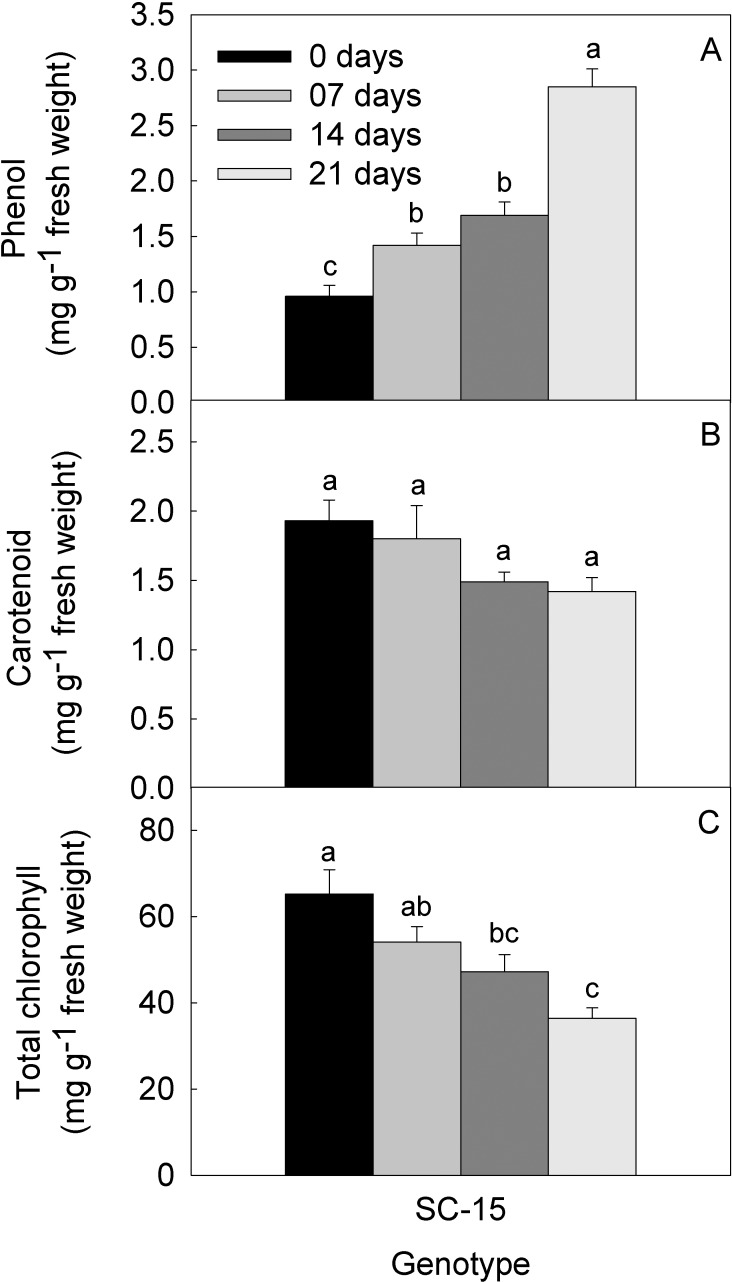
**Concentration of** (a) phenol (b) carotenoid (c) total chlorophyll, in muskmelon leaves under 0 (well-watered), 7, 14 and 21 days of water-deficit. The results are mean ± SE of triplicate measurements, means followed by the same letter are not significantly different (P ≤ 0.05), according to Duncan’s multiple range test.

The content of phenol was measured in plant during various stages of water-deficit; exhibited an increasing trend (Fig 2A). In well-watered plants phenol content was 0.96 (mg g^-1^ FW), while content of phenol increased by 48% and 76% after 7 and 14 DWD. The increase was rather drastic after 21 DWD as it increases by 196%, compared with control, respectively. Carotenoid levels were monitored in the muskmelon leaves after 7, 14 and 21 DWD. Carotenoid level declined with an increase in DWD. The decrease in carotenoid pigments was 5.6%, 22.8% and 26.4% after 7, 14 and 21 DWD, in comparison to well-watered plants (Fig 2B), respectively. The total chlorophyll content showed a declining trend after 7, 14 and 21 DWD, as its content reduced by 17%, 39% and 62%, respectively, in comparison to well-watered plants (Fig 2C).

### Changes in gene expression those linked proteins showed significant changes in abundance

A total of eight protein species belong to various cellular and molecular group along with significant changes in their abundance were selected for their respective gene expression study through RT-qPCR (Table 1). Out of these, six protein genes (RNA pseudouridine synthase 7, chromoplast-specific carotenoid-associated protein, dihydroflavonol-4-reductase, 28 kDa ribonucleoprotein, cytochrome P450 and NADPH adrenodoxin oxidoreductase) matched at the mRNA level with their protein expression level. However, level of transcription and translation varied under different drought stress condition. Moreover, our results also revealed two genes encoding protein (Ras-related protein RABD1 and MADS-box transcription factor), displaying different protein and mRNA levels under different drought stress condition, as their protein expression increases, while the corresponding gene expression level decreases.

**Fig 3:**
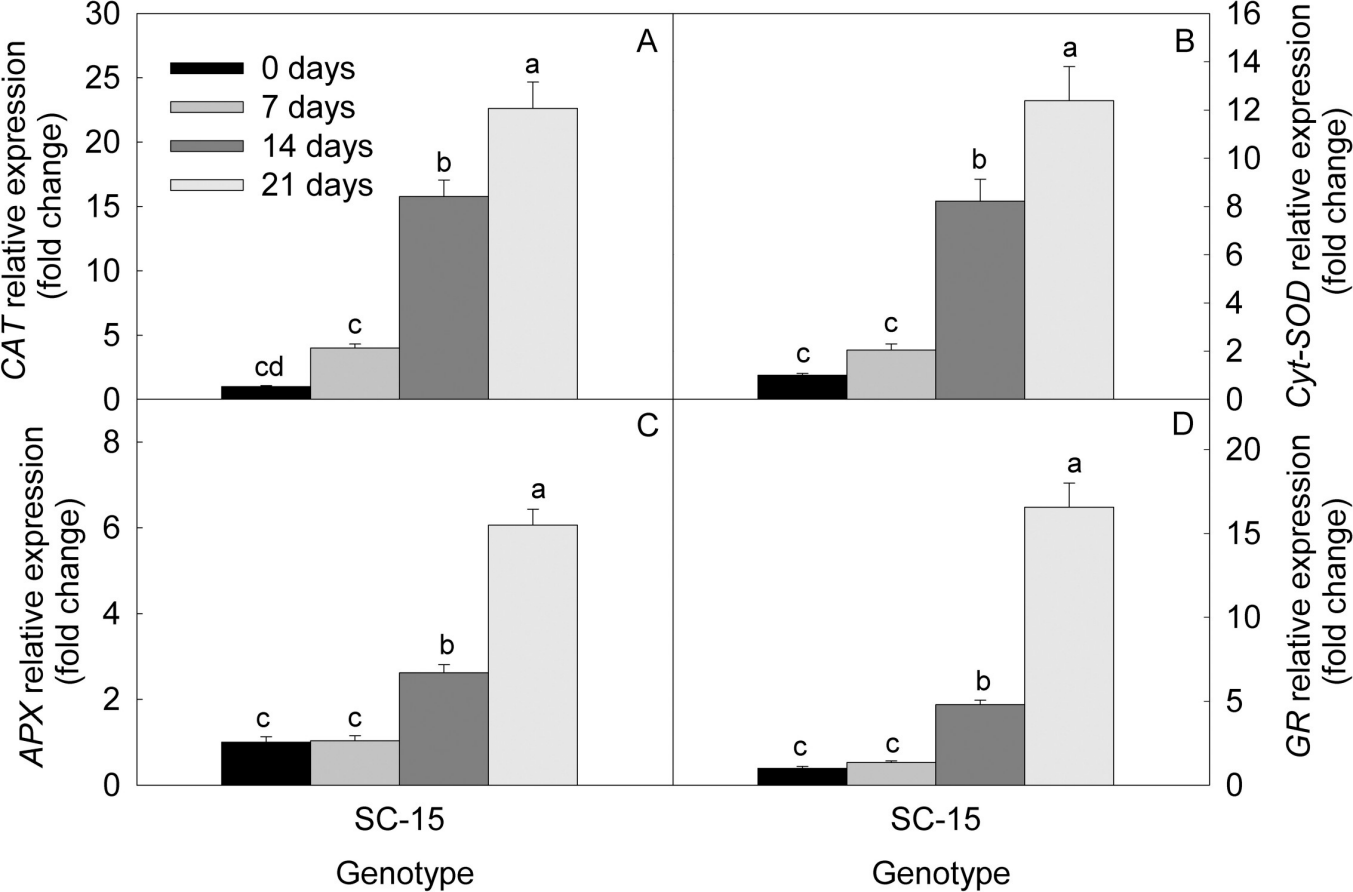
Relative expression of antioxidative enzyme genes. (A) *CAT* (AY274258.1), (B) *Cyt-SOD* (EU407180.1), (C) *APX* (FJ890985.1), and (D) *GR* (GU248528.1), in muskmelon leaves under 0 (well-watered), 7, 14 and 21 days of water-deficit. The results are mean ± SE of triplicate measurements, means followed by the same letter are not significantly different (P ≤ 0.05), according to Duncan’s multiple range test.

Increase in days of water-deficit results in to induced expression of *SOD* (EEU407180) in muskmelon leaves. *SOD* expression increases by 2, 8 and 12 fold after 7, 14 and 21 DWD, respectively, compared with control plants. Real time PCR analysis showed enhanced expression of *CAT* (AY274258.1), *APX* (FJ890985.1) and *GR* (GU248528.1) in drought stressed muskmelon plant leaf after 7, 14 and 21 DWD. Transcript level of *CAT* was increased by 4, 16 and 23 fold after 7, 14 and 21 DWD compared to well-watered plant. However, under similar condition 1, 2.6 and 6.1 fold over-expression recorded for *APX* and 1.3, 4.8 and 16.6 fold over-expression in case of *GR*, compared with well-watered plants (Fig 3A–3D).

### 2D analysis of proteins

**Fig 4:**
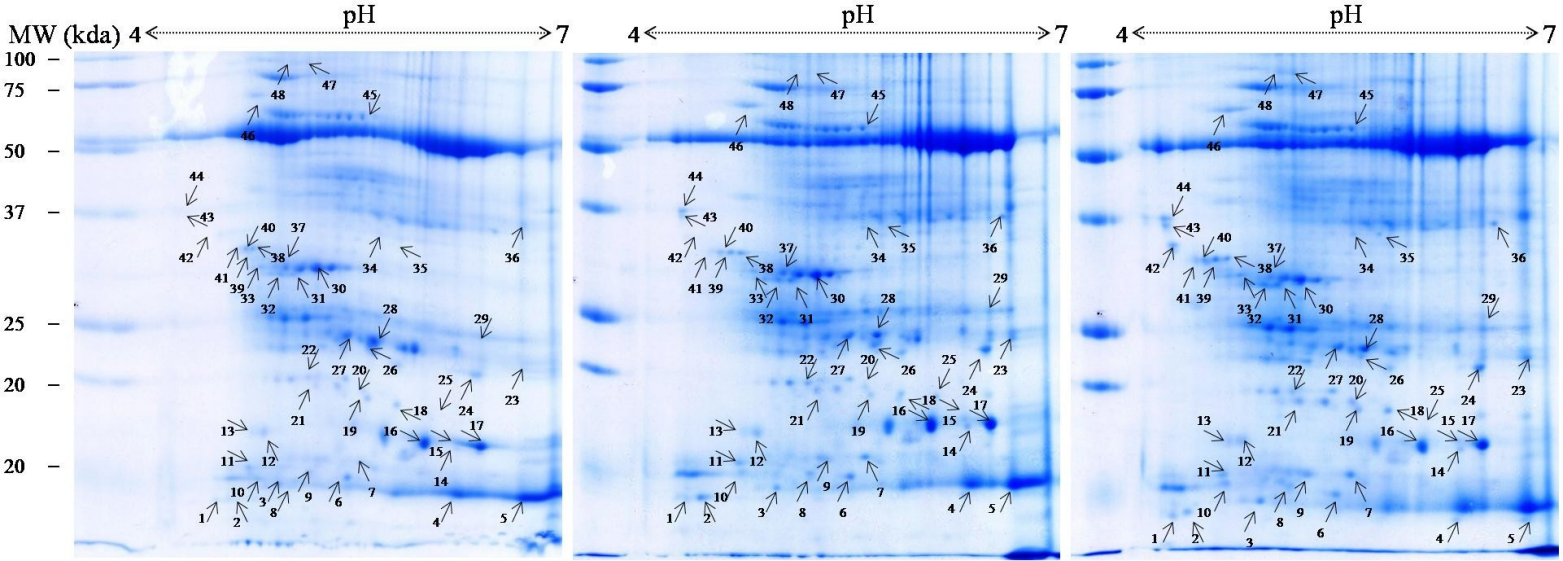
A 2-D gel electrophoresis analysis of muskmelon leaf proteins of drought tolerant SC-15. Subjected to 0 (well-watered), 7 and 14 days of water-deficit (left to right). A total of 250 mg of proteins were extracted and separated by 2-D gel and visualized with coomassie brilliant blue (CBB) stain.

**Fig 5:**
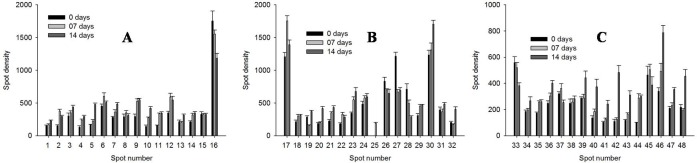
Changes in spot density of the identified proteins in muskmelon leaves. Under 0 (well-watered), 7 and 14 days of water-deficit condition.

To understand the water-deficit response mechanism of muskmelon at proteome level, the expression pattern of proteins was determined using proteomic approach. Extraction was carried out for the total soluble proteins of muskmelon leaves that have been exposed to 7 and 14 DWD along with well-watered plants. Two-dimensional gel images of muskmelon leaves were observed after the completion of water-deficit treatment, using IPG strip of 11 cm (pH 4–7) and 12% SDS-PAGE. Spot detection on CBB stained gels (Fig 4), revealed total 265 reproducible protein spots, which were identified using PDQuest software (Bio-Rad, USA). A comparative analysis exhibited that 48 protein spots were differentially expressed (P≤ 0.05). Total spots influenced by 7 and 14 DWD treatments were 48 (37 up-regulated, 10 down-regulated, 01 new) and 47 (38 up-regulated, 09 down-regulated), and one newly expressed (spot no, 25) identified as MADS-box transcription factor, respectively, (Fig 5A–5C, Table 3).

**Table 3:** Drought induced differentially-expressed proteins under 7 and 14 days of water-deficit in muskmelon leaf, identified using MALDI-TOF/TOF/MS analysis.

S.No	Spot No.	Homologue proteins	Species	Accession number/ GI	Mr/pI Theoretical	Sequence coverage (%)	Mascot score	Biological process/molecular function	Functional category	*Fold change (7/14 days)	*7 /14 days
1	14	Ribosomal protein L36	*Cystopterisprotrusa*	AJE61594 748763081	16.80/6.45	54	21	Translation	Translation	1.50/1.65	↑/↑
2	17	Ribosomal protein S7	*Epipogiumroseum*	AII40866 670800465	17.95/6.57	35	43	Translation	Translation	1.45/1.15	↑/↑
3	18	Putative ribosomal protein S4, partial	*Pinus sylvestris*	ACJ70414 215809414	18.85/6	29	20	RNA binding/translation	Translation	1.40/1.46	↑/↑
4	22	Putative ribosomal protein S4	*Pinus sylvestris*	ACJ70414 215809414	20/5.22	43	17	RNA binding, translation	Translation	1.80/1.56	↑/↑
5	31	Translation initiation factor 1	*Selaginellauncinata*	BAE53390 82949456	29/5.28	38	22	Protein biosynthesis	Translation	0.93/1.23	↓/↑
6	24	PSII phosphoprotein	*Panax ginseng*	ABD73301 89475538	22.20/6.55	62	50	Photosynthesis	Photosynthesis	1.20/1.24	↑/↑
7	30	Oxygen-evolving enhancer protein 1	*Vitis vinifera*	XP_002274796 225468761	31.70/5.25	71	69	Photosynthesis	Photosynthesis	1.06/1.38	↑/↑
8	33	Oxygen-evolving enhancer protein 1	*Cucumis sativus*	XP_004141946 449448384	29.24/5.38	65	67	Photosynthesis	Photosynthesis	0.93/0.68	↓/↓
9	4	RNA pseudouridine synthase 7	*Arabidopsis thaliana*	PUS7_ARATH 75264240	8/6.40	49	45	RNA binding	Nucleotide biosynthesis	1.85/2.25	↑/↑
10	8	Ribose-phosphate pyrophosphokinase 4	*Spinacia oleracea*	KPRS4_SPIOL 62286957	15.60/5.35	51	37	Nucleotide biosynthesis	Nucleotide biosynthesis	1.21/1.07	↑/↑
11	9	Polypyrimidine tract-binding protein homolog 1	*Arabidopsis thaliana*	PTBP1_ARATH 75336151	16/5.40	54	33	mRNA processing	Nucleotide biosynthetsis	1.79/1.86	↑/↑
12	27	Diacylglycerol kinase 5-like	*Oryza brachyantha*	XP_006644806 573914273	23.82/5.56	63	37	ATP Binding, nucleotide binding	Nucleotide biosynthesis	0.55/0.57	↓/↓
13	44	Plastid lipid-associated protein 3	*Cucumis sativus*	XP_004148751 449462043	38.20/4.16	91	76	Oxidoreductase	Stress response	2.92/3.05	↑/↑
14	41	Dihydroflavonol-4-reductase	*Aegilops speltoides*	BAH36915 226069382	30.15/4.51	83	51	Catalytic activity	Stress response	1.23/2.34	↑/↑
15	40	Chromoplast-specific carotenoid-associated protein	*Cucumis sativus*	XP_004134784 449434000	29.95/4.53	85	89	Carotenoid sequestration within chromoplasts	Stress response	1.37/2.76	↑/↑
16	39	Chromoplast-specific carotenoid-associated protein	*Cucumis melo*	XP_008440050 659079054	29.97/4.70	78	49	Carotenoid sequestration within chromoplasts	Stress response	1.01/1.55	↑/↑
17	38	Peroxidase	*Triticum urartu*	EMS52001 473994695	29.95/4.76	81	59	Peroxidase activity, response to oxidative stress	Stress response	1.04/1.14	↑/↑
18	6	NAC transcription factor	*Zea mays*	ADX60089/ 323388569	15.55/5.40	43	45	Transcription regulation	Stress response,transcription	1.33/1.12	↑/↑
19	36	Putative WRKY transcription factor 70	*Triticum urartu*	EMS49450/ 473907662	38.30/6.63	52	50	Transcription factor activity, sequence-specific DNA binding	Stress response, transcription	0.62/0.75	↓/↓
20	28	SRF-type transcription factor protein	*Medicago truncatula*	AES79095 657377718	22.44/5.68	72	49	Transcription regulation	Stress response transcription	0.69/0.41	↓/↓
21	21	S-RNase	*Prunus speciosa*	BAF56273 144905315	19.25/5.21	66	40	RNA binding	Transcription regulation	1.56/1.93	↑/↑
22	34	NAC domain protein	*Arabidopsis lyrata* subsp. *lyrata*	XP_002874324 297812881	36.38/5.52	71	23	Transcription regulation	Transcription regulation	1.07/1.45	↑/↑
23	25	MADS-box transcription factor	*Lacandoniaschismatica*	ADC53557 288561771	17/6.20	73	54	Transcription regulation	Transcription regulation	-	
24	5	Metallothionein-like protein 1	*Prunus avium*	MT3_PRUAV 3334263	7.75/6.75	54	30	Metal ion binding	Metabolism	1.34/2.85	↑/↑
25	7	SPX domain-containing protein 3	*Oryza sativa* subsp. *indica*	SPX3_ORYSI 306756002	16.25/5.70	60	24	Cellular response to phosphate starvation	Metabolism	1.28/1.72	↑/↑
26	13	GA biosynthesis enzyme	*Chrysanthemum morifolium*	ACC68671 184161279	18/4.75	50	37	Oxidoreductase activity	Metabolism	0.96/1.46	↓/↑
27	15	Predicted protein	*Micromonaspusilla*	XP_003063654 303288732	18.20/6.42	39	35	Rhamnose metabolic process	Metabolism	0.98/0.98	↓/↓
28	19	Pantothenate kinase	*Gossypium arboreum*	KHG07019 728827055	18.87/5.82	28	20	Kinase activity	Metabolism	0.56/1.29	↓/↑
29	32	Probable alpha, alpha-trehalose-phosphate synthase	*Oryza brachyantha*	XP_006659396 573954294	75/5.25	37	35	Catalytic activity, trehalose biosynthetic process	Metabolism	1.22/1.32	↑/↑
30	37	Nitrate reductase	*Schiedeastellarioides*	ADG43082 295844200	31.70/4.84	48	22	Oxidoreductase activity	Metabolism	0.89/0.70	↓/↓
31	26	Ribulose bisphosphate carboxylase/oxygenase activase	*Oryza sativa Indica Group*	ABR26165 149392725	21.85/5.90	52	49	ATP Binding	Carbohydrate metabolism	0.84/0.78	↓/↓
32	47	Ribulose-1,5-bisphosphate carboxylase/oxygenase large subunit	*Podococcusbarteri*	CAJ33779 90968286	97.25/5.42	51	49	Carbon fixation	Energy	1.11/1.69	↑/↑
33	3	Non-symbiotic hemoglobin 4	*Oryza sativa* subsp. *japonica*	HBL4_ORYSJ 22001644	15.20/4.80	57	36	Metal ion binding, electron transfer	Energy	1.16/1.45	↑/↑
34	46	Cytochrome P450 CYP93B25	*Salvia miltiorrhiza*	AJD25217 745791017	58.95/4.77	52	50	Oxidoreductase	Energy	1.12/0.79	↑/↓
35	48	NADPH adrenodoxin oxidoreductase	*Zea mays*	ACG45856 195652777	97.42/5.18	78	45	Oxidation-reduction process	Energy metabolism	0.90/2.10	↓/↑
36	45	ATP synthase CF1 alpha subunit	*Gunneramanicata*	ADD31507 290490200	61.45/5.63	86	58	ATP synthesis, hydrogen ion transport	Energy metabolism	1.09/0.84	↑/↓
37	10	Ras-related protein RABD1	*Arabidopsis thaliana*	RABD1_ARATH 75338904	15.85/4.75	74	42	Small GTPase mediated signal transduction	Transport, signal transduction	1.94/2.93	↑/↑
38	16	Calmodulin-like protein 7	*Arabidopsis thaliana*	CML7_ARATH 75335243	17.90/6.15	75	38	Calcium ion binding	Signal transduction	0.89/0.68	↓/↓
39	29	Glycolipid transfer protein-like	*Oryza sativa* subsp. *japonica*	NP_001047468 115447377	25.45/6.55	77	57	Glycolipid binding, glycolipid transporter activity	Transport	1.48/1.52	↑/↑
40	23	Peptidyl-prolyl cis-trans isomerase	*Sorghum bicolor*	XP_002444806 242080075	22.90/6.75	69	54	Protein folding	Protein folding	1.60/1.96	↑/↑
41	42	28 kDa ribonucleoprotein	*Cucumis melo*	XP_008464495 659129034	32.85/4.28	64	82	Nucleic acid binding	Nucleic acid binding	1.16/4.41	↑/↑
42	35	MYB5	*Dendrobium* sp.	AAO49414 28628955	36.35/5.88	68	25	DNA binding	DNA binding	1.49/1.50	↑/↑
43	43	Putative uncharacterized protein	*Chlorella variabilis*	XP_005850377 552841693	36.15/4.25	90	68	-	Unknown	1.41/2.66	↑/↑
44	12	Uncharacterized protein	*Genliseaaurea*	EPS64397 527194982	18.10/4.83	86	60	-	Unknown	1.70/1.57	↑/↑
45	20	Uncharacterized protein	*Arabisalpina*	KFK43912 674251147	19/5.75	59	42	-	Unknown	1.06/2.22	↑/↑
46	1	Not identified								1.12/1.52	↑/↑
47	2	Not identified								2.48/1.93	↑/↑
48	11	Not identified								2.17/2.24	↑/↑

*Compared with control

Differentially expressed and identified 42 proteins were associated with different cellular process. Detailed information of differentially expressed proteins with relevant information is provided in Table 3, after exploring the information with the help of PMF, MASCOT search and uniprot database. Related parameter included spot number, protein, species, theoretical PI, molecular weight, GI, accession number, MASCOT SCORE, peptide number, sequence coverage, biological process/ molecular function they mediate. Hierarchical clustering of expression profiles were also presented to show the grouping pattern of proteins, in total six clusters the proteins has been grouped based on their level of expression under control and 07 and 14 DWD (S1 Fig).

**Fig 6:**
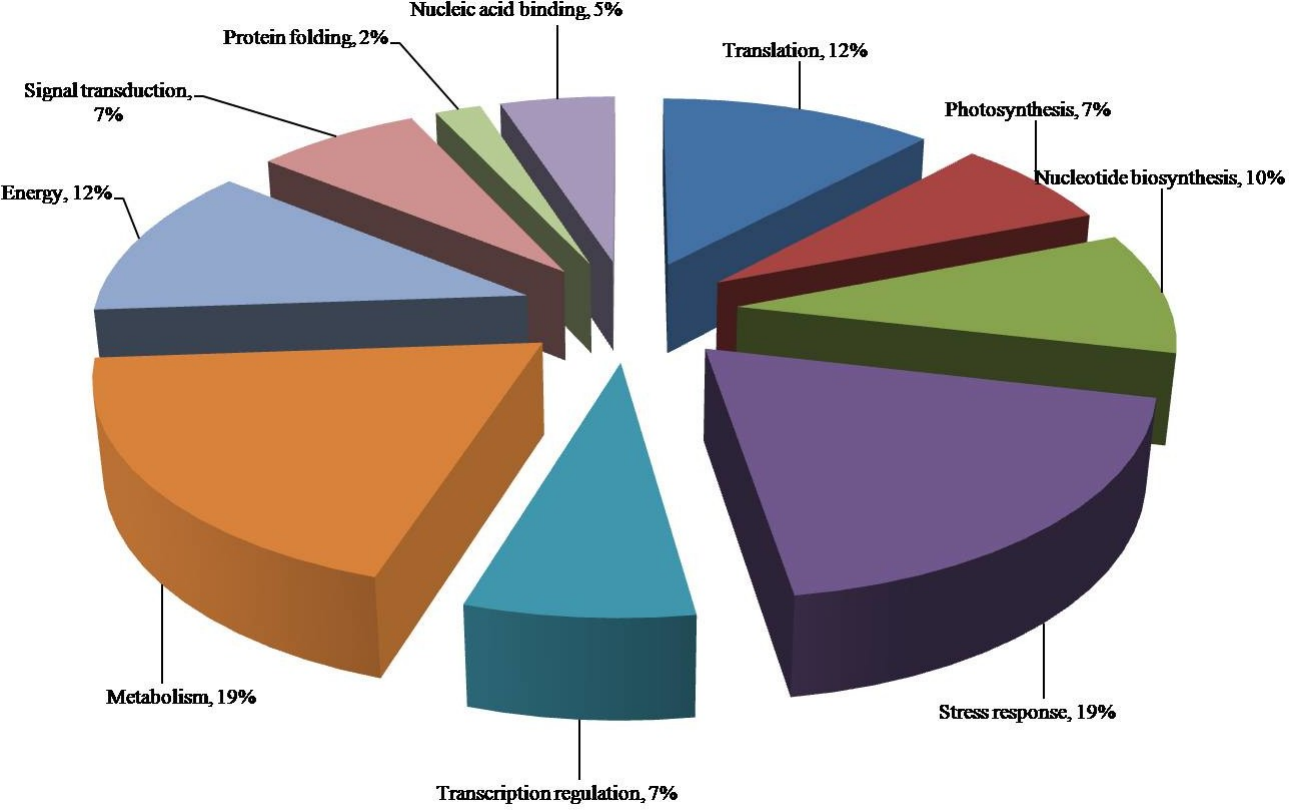
Functional distribution of identified differentially accumulated proteins in muskmelon. Under 0 (well-watered), 7 and 14 days of water-deficit, categorized based on their gene ontology annotations.

The biological functions of differentially expressed proteins includes, translation, photosynthesis, nucleotide biosynthesis, response to stress and response to abiotic stimulus, transcription regulation, metabolism, energy metabolism, signal transduction, transport, protein folding and nucleic acid binding (Fig 6).

## Discussion

Muskmelon is an economically important crop widely consumed around the world, mostly in arid and semi arid regions. Similar to other arid and semiarid dry land crops, drought stress is a major limitation of agriculture production which also reduces muskmelon yield and quality markedly.

### Morpho-physiological and biochemical changes

A better penetrated and prolific root system was establish to be connected with improved tolerance of terminal drought in muskmelon [13], similarly in present experiment enhance root length and decrease shoot length was observed under drought stress. In same condition, *Fv*/*Fm* were negatively affected, may be due to alteration in photochemical conversion efficiency [36]. Declined photosynthetic capability of the plants under drought stress could had been contributed due to damage to PSII [13]. Better water withholding capacity is the physiological characteristics of plants which exhibit better tolerance to water-deficit. With increased DWD the tissues retaining water decreases however it was lower in compared to susceptible lines as reported earlier [13], this results into increased dry and fresh weight ratio. Similar observation were noted in present experiment as dry and fresh weight ratio of shoot, leaf and root increases, which peaked at maximum after 21 DWD.

Enhanced accumulations of phenolic compounds under abiotic stress condition are the biochemical adaptability of plants to destroy free radicals [37]. As reported earlier, in the present study SC-15 provides better cell integrity even under 21 DWD, which is supported with the increased concentration of phenol. Contraction in total chlorophyll and carotenoid content was reported [38], under water-deficit condition. Such lowering in the level of chlorophyll may be endorsed to a reduction in chlorophyll biosynthesis or an enhancement in chlorophyll degradation, similar results were also observed in our study.

### Gene expression

A weak correlation observed between mRNA and abundance of protein which may be due to post-transcriptional, translational and post-translational systems in the course of mRNA translation and degradation of protein. When plants faces environmental stresses CAT, SOD, APX and GR protect plants from oxidative damage [38]. Similar reports were also published in bottle gourd and cucumber [39, 40]. In the present experiment, transcript level of *CAT* enhanced after 7, 14 and 21 DWD. Similarly tolerance to drought in transgenic rice plants due to over-expression of *OsMT1a*, was reported [41]. An increased drought tolerance in *Cu/Zn-SOD*, *APX* and *Mn-SOD* transgenic tobacco were reported [42]. A similar finding also noted in present experiment where expression of *SOD*, *APX*, and *GR*, enhanced with increase in DWD. Better tolerance capability under water-deficit performed by SC-15, may be due to the enhanced expression of these antioxidant enzyme genes.

### Differential expression of proteins involved in translation, photosynthesis and nucleotide biosynthesis

Enhanced expression of translation related protein was up to 14 DWD. However, ribosomal protein S7 and translation initiation factor 1 expression decreases after 7 DWD up to 14 DWD (Table 3). Enhanced expressions of ribosomal proteins L36 were reported under drought stress[43]. Differential expression of ribosomal protein S4 was noted during Cd, NaCl and their combined treatment in *S*. *oleracea* [44]. Reports suggest that, the drought-induced regulation of translation related proteins based on the plant species and the duration of the water-stress [45, 46, 47, 48].

An oxygen-evolving enhancer protein 1 was down-regulated up to 14 DWD. Similarly, Bagheri *et al*. [44] reported highly down regulated expression of this protein under Cd and Cd + NaCl, stress, respectively. Reduction in various phosphoproteins has been reported under drought stress, similarly in current experiments the levels of PSII phosphoprotein were lower under well-watered conditions compared to water-deficit.

For the most part, posttranscriptional change in cellular RNAs are catalyzed by enzymes pseudouridine synthases, catalyzes the site-specific isomerization of uridine residues which is a part of an RNA chain, employing both sequence and structural information to get site specificity. The level of RNA pseudouridine synthase 7 enhanced after 7 and 14 DWD, compared with well-watered plants, which indicates un-interfered site-specific isomerization of uridine residues. Ribose-phosphate pyrophosphokinase 1 (PRPS1) catalyzes the synthesis of phosphoribosyl pyrophosphate (PRPP), one of the intermediate required for producing nucleotides, including pyridine nucleotide cofactors NAD and NADP and histidine and tryptophan [49]. In the present study, water-deficit enhanced the availability of PRPS1; however, a small reduction was observed after 7 DWD to 14 DWD. Similarly, Bagheri *et al*. [44] reported increased expression of PRPS1 under cadmium stress. The polypyrimidine tract-binding protein (PTB) homolog 1 level increased after 7 and 14 DWD, 50-kD PTB protein, RBP50, which is the core of protein and RNA components of a plant phloem ribonucleoprotein (RNP) complex.

### Differential expression of proteins involved in stress response and transcription regulation

Accumulation of plastid lipid associated proteins in fibrillar-type chromoplasts under abiotic stress condition were reported, for example in ripening pepper fruit and leaf chloroplasts from Solanaceae plants [50]. Increase in level of plastid lipid-associated protein 3 during initial stress exposure indicated mild water-deficit doesn’t affect the synthesis of this protein while the water-deficit beyond a limit reduces synthesis. Hu *et al*. [51], functionally characterized dihydroflavonol-4-reductase and suggested that it act in purple sweet potato anthocyanin biosynthesis which indicates the straight proof function of anthocyanins under abiotic stresses. Our finding also supports as the level of Dihydroflavonol-4-reductase in muskmelon leaf increased under elevated water-deficit condition, which may favors the better tolerance of SC-15 against water-deficit. NAC transcription factors plays major role in drought stress response in plants [51], enhanced expression of NAC gene under drought. Similarly in our study, NAC transcription factor was markedly up-regulated under water-deficit condition. WRKY transcription factor family involvement in plants under stress suggests that plant establish their defense system to protect with such situations [52]. In our study the putative WRKY transcription factor 70 expression reduced at 7 and 14 DWD, however compared to level at 7 days the level increases at 14 DWD, in spite of this the level was lower compared to well-watered plants. Bagheri *et al*. [44], reported up-regulation of WRKY transcription factor under cadmium stress and reduction when spinach plants were exposed to salt stress.

Reports suggested that MADS domain proteins tie to particular DNA components known asCArG boxes, most familiar is the serum response element (SRE or SRF)-type CArG box, which has the consensus CC[A/T]_6_GG. In our studies the level of SRF-type transcription factor protein increases at 7 DWD while it reduces lower than the control plants at 14 DWD, indicates during initial stress exposure enhanced protein level helps plants to adopt against water-deficit. Involvement of NAC domain proteins in various developmental processes of plants has been concerned. Specifically, several NAC domain proteins found to be involved in plant abiotic stresses defense responses. NAC domain protein level in present experiment enhances under water-deficit. Suggests the elevated expression of this protein in SC-15 helps to resist against water-deficit. MADS-box transcription factors observed to be up-regulated in current study, which is a major development regulator in flowering plants.

### Metabolism related proteins abundance in response to drought stress

To increase the phosphorus (P) use efficiency plants developed a wide spectrum of mechanisms. In plants to maintain the Pi homeostasis, studies at molecular level suggested the involvement of several proteins carrying the SPX domain. Increase in the level of this protein in muskmelon leaf under water-deficit condition, exhibits maintained phosphorus homeostasis in muskmelon plants under water-deficit condition.

In the regulation of higher plants developmental and various growths processes, the involvement of large family of tetracyclic diterpenoid phytohormones were reported [53]. Initially up to 7 DWD a slight decrease noted, however, after 7 to 14 DWD, an elevation in the abundance of this protein noted in the current study. Pantothenate kinase phosphorylates pantothenate (vitamin B5), to form 4'-phosphopantothenate employing one molecule of adenosine triphosphate (ATP). Accordingly, pantothenate kinase one of the major regulatory enzyme in the CoA biosynthetic pathway [54], in present experiment an increased measured in the level of panthotenate kinase in muskmelon leaf under elevated water-deficit condition, suggest maintained TCA cycle and fatty acid metabolism, even under water-deficit exposure.

### Energy related proteins abundance

Significant influence of water-deficit on plants energy metabolism has been established. Ribulose-1, 5-biphosphate carboxylase (RuBisCO) is one of the most important enzyme in plant leaves for carbon metabolism. 3-phosphoglyceric acid is produced when CO_2_ interact with ribulose-1, 5-biphosphate (RuBP) by RuBisCO during photosynthesis. Under stress condition a vital role is played by RuBisCO and ATPase beta to sustain chloroplast and whole cell function of plant [55]. In present findings, RuBisCO large subunit observed to be increased significantly, the finding follows similar reports in sugarcane under drought stress [56, 57] and tolerant variety of sunflower when exposed to drought stress [58]. Maintained and elevated expression of this protein in muskmelon leaf under water-deficit may be responsible for the better tolerance of SC-15 genotype under water-deficit condition. Biochemical investigations have highlighted P450s acting on fatty acids (FAs): can be enhanced by biotic and abiotic stress at the transcriptional level. P450s able to produced oxidized FA have been recognized and characterized from a variety of plant species [59]. Supporting the above reports in our study the level of Cytochrome P450 enhanced after 7 and 14 DWD. NADPH: adrenal ferredoxin adrenodoxin reductase, a FAD-containing flavoprotein an important constituents of electron transport system for the cytochrome P-450-dependent hydroxylation [60]. In our study the protein NADPH adrenodoxin oxidoreductase decreases during initial stress exposure of water-deficit up to 7 DWD, however, then it increases after 14 DWD.

### Abundance of protein involved in protein folding, nucleic acid binding transport and signal transduction

Calmodulins, calmodulin-like proteins, calcineurin B-like and CaM-binding proteins are Ca^2+^ sensing protein families which plant possess and affected under stress condition [61]. In present study, calmodulin-like proteins7 were down-regulated under water-deficit condition. At 7 DWD a 24 kDa protein glycolipid transfer protein level decreases, this protein is involved in in vitro transfer of glycolipids from one bilayer membrane to another [62], though glycolipid transfer protein concentration rises after 14 DWD.

In oligopeptides and proteins cis/trans isomerization of the peptidyl-prolyl peptide bond iscatalyzed by peptidyl-prolyl cis/trans isomerases (PPIases), a critical actionduring the progression of folding of protein which is crucial for the generation of functional proteins. Increased abundance of Peptidyl-prolyl cis-trans isomerase in muskmelon leaf under water deficit, suggest a non-interrupted isomerization of the peptidyl-prolyl peptide bond in oligopeptides and proteins. Nuclear-encoded 28 kDa ribonucleoproteins (RNPs) is vital for plastid mRNA 3’ end processing and constancy in chloroplasts [63], was increased very slowly during 7 DWD, but a rapid enhancement in this protein level noted at 14 DWD. The increase in MYB5 protein was very high during initial stress exposure, while the MYB5 level was almost constant at 7 and 14 DWD. At transcriptional level many important biological process were controlled by change in gene expression. The group of MYB transcription factor is extended and found to be participating in response to various biotic and abiotic stresses.

## Conclusions

The finding enhances our knowledge about the alteration in protein level under drought stress that could be used as marker for drought stress, it is the beginning stage for more proteomic, molecular and physiological investigations to comprehend mechanism affecting drought stress in muskmelon genotype. Out of the identified 265 protein spots 48 were statistically significantly differentiate in their abundance in SC-15 genotypes under different water-deficit condition, namely 7 days and 14 days of water deficit compared to their abundance under control condition. Present investigation propose that muskmelon has adjusted to water- deficit utilizing avoidance mechanism, that incorporates decrease in growth enabling plants to maintain cellular homeostasis, also the enhanced expression of antioxidant enzyme gene, that reduces the level of free radical generated under drought stress. Drought induced proteome alteration, showed muskmelon genotype SC-15 does not rely on a particular proteins or any individual pathway, instead of this a complex response is generated. To protect or avoid from the stresses, plants induces new proteins generally, however, sometimes it stops the synthesis of others. In coming years study must be focused on proteomic approaches based on comparative and advanced functional analysis of the differentially expressed proteins for inclusive information and engineering approaches to enhance plant tolerance against drought stress.

## Supporting information

S1 Fig Hierarchical clustering of expression profiles. The differentially expressed 48 proteins were grouped into 06 clusters based on their expression profiles. Each square represents a single spot on a single gel, with each row representing a single spot across all of the gels in the experiment, and each column representing all of the spots on a single gel.
(TIF)

S1 Table Nucleotide sequences of primers used in quantitative real-time PCR (qRT-PCR). (DOCX)
