# Supplementary figures and images for: Drought mediated physiological and molecular changes in muskmelon (Cucumis melo L.)
Source: PLoS One. 2019 Sep 24;14(9):e0222647. doi: 10.1371/journal.pone.0222647 (PMC6759176; doi:10.1371/journal.pone.0222647)

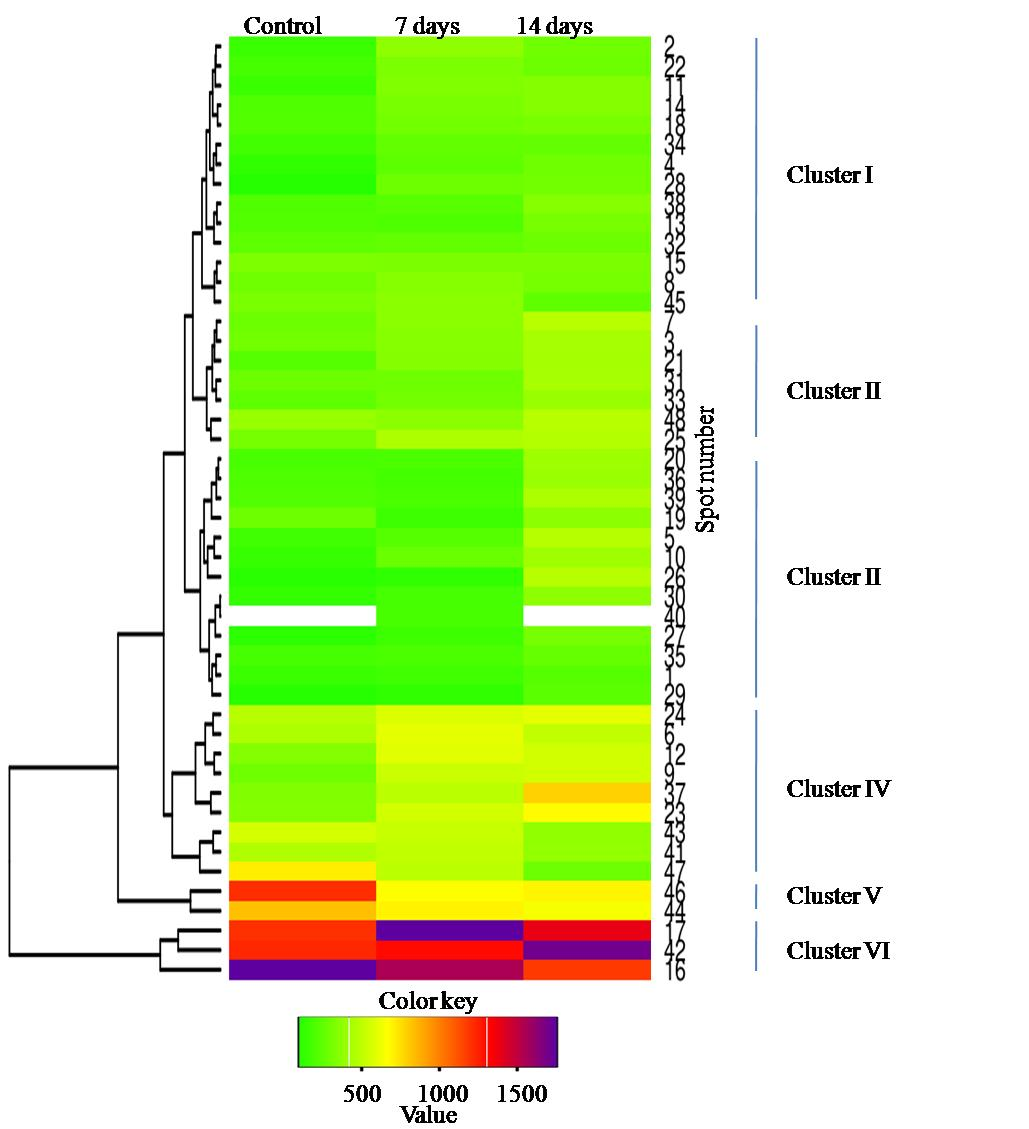

Supplement: S1 Fig — The differentially expressed 48 proteins were grouped into 06 clusters based on their expression profiles. Each square represents a single spot on a single gel, with each row representing a single spot across all of the gels in the experiment, and each column representing all of the spots on a single gel. (TIF) [file pone.0222647.s001.tif]
