# Supplementary material for: Drought mediated physiological and molecular changes in muskmelon (Cucumis melo L.)
Source: PLoS One. 2019 Sep 24;14(9):e0222647. doi: 10.1371/journal.pone.0222647 (PMC6759176; doi:10.1371/journal.pone.0222647)
Supplement: S1 Table — (DOCX) [file pone.0222647.s002.docx]

**S1 Table 1. DNA sequences of PCR primers used in quantitative real-time PCR (qRT-PCR).**

| **Unigene ID** | **Genes** | **Forward primer sequence** | **Reverse primer sequence** |
| --- | --- | --- | --- |
| AY274258.1 | *CAT* | TCCACTGGAAACCAACTTGCGG | ACCAATCTCCCCACCGGCTGTA |
| EU407180.1 | *Cyt-SOD* | AAGCCCGGGCTTCATGGATTCC | TGAACCACAACAGCCCTTCCGA |
| FJ890985.1 | *APX* | ACAGGACAAACCCGAGCCACCA | CGTTCCTTGTGTGCCCTACCCA |
| GU248528.1 | *GR* | ACACGTCGTCGTTCAATCCAA | TGCGCCAAGGACCTTTTGT |
| AY859055 | *Actin* | CCCTGGTATTGCAGACAGGA | ACATCTGCTGGAAGGTGCTT |
